# The role of hydrogen bonding in the incommensurate modulation of *myo*-inositol camphor ketal

**DOI:** 10.1107/S2052520620015929

**Published:** 2021-01-22

**Authors:** Viktor Savic, Felix Eder, Christian Göb, Marko D. Mihovilovic, Christian Stanetty, Berthold Stöger

**Affiliations:** a Institute of Applied Synthetic Chemistry, TU Wien, Getreidemarkt 9/163, A-1060, Vienna, Austria; b Institute of Chemical Technologies and Analytics, TU Wien, Getreidemarkt 9/164, A-1060, Vienna, Austria; c Rigaku Europe SE, Hugenottenallee 167, 63263, Neu-Isenburg, Germany; d X-Ray Center, TU Wien, Getreidemarkt 9/164, A-1060, Vienna, Austria

**Keywords:** incommensurate modulation, hydrogen bonding, X-ray diffraction, sugar chemistry

## Abstract

*myo*-Inositol-2,3-d-camphor ketal crystallizes as an incommensurate structure with the *C*2(0σ_2_½) superspace group symmetry [σ_2_ = 0.1486 (3) at 100 K]. The bornane and *myo*-inositol moieties aggregate in distinct layers extending parallel to (001). The *myo*-inositol rings are connected by a complex hydrogen-bonding network extending in two dimensions, which is disordered in the basic structure and (mostly) ordered in the actual modulated structure. The domains of definition of the H atoms in internal space were derived by chemical reasoning and modeled with crenel functions. By tracing the hydrogen bonding, distinct chains, which are periodic in the [100] direction, are identified. These chains possess one of two possible orientations with respect to the hydrogen bonding. The incommensurate modulation is characterized by a non-periodic succession of the two chain orientations in the [010] direction. On heating, the σ_2_-component of the modulation wave vector decreases from σ_2_ = 0.1486 (3) at 100 K to σ_2_ = 0.1405 (6) at 430 K, which means that the periodicity of the modulation wave increases. No order–disorder phase transition was evidenced up to the melting point (with decomposition).

## Abstract 2

The incommensurate modulation of *myo*-inositol-2,3-d-camphor ketal is due to a complex diperiodic hydrogen-bonding network.

## 1. Introduction   

Hydrogen bonding is a common source of order–disorder phase transitions, whereby the hydrogen-bonding network is disordered at high temperatures and ordered at low temperatures. An important example is the potassium dihydrogenphosphate family of compounds, whose members are ferroelectric below and paraelectric above the order–disorder phase transition temperature (Schmidt, 1987 ▸).

Incommensurately modulated structures often are intermediate phases, which exist in a temperature range between ordered commensurately modulated phases and disordered non-modulated phases (Cummins, 1990 ▸). The transition between the incommensurate and the disordered phases can be considered as being of the order–disorder type. In this special case the group–subgroup relation is of index ∞, since the dimensionality of the translation lattice is reduced in the incommensurate phase.

When these facts are combined, it is unsurprising that ordering of the hydrogen-bonding network has been reported as the crucial feature of numerous incommensurately modulated structures in all classes of compounds from inorganic to organometallic and organic. For example, (non-classical) hydrogen bonding plays a crucial role in the incommensurate modulation of thiourea (Gao & Coppens, 1989 ▸), which is one of the most intensely studied incommensurately modulated structures. Another well known incommensurate structure involving ordering of hydrogen bonds is ferroelectric bis(*n*-propylammonium)tetrachloro­manganate(II) (Depmeier, 1986 ▸). More recently, hydrogen bonding has been identified as the main driving force in the order–disorder transition of β-[Zn-(pydcH)_2_]·3H_2_O, where pydcH is mono-deprotonated pyridine-2,6-dicarboxylic acid, leading to an incommensurately modulated low-temperature structure (Tabatabaee *et al.*, 2018 ▸). Likewise, the pivotal role of hydrogen bonding in the incommensurate modulation of [CH_3_NH_3_][Co(COOH)_3_] has lately been elucidated using neutron diffraction (Canadillas-Delgado *et al.*, 2019 ▸). Order–disorder phase transitions have been described for the organic salt hexamethylenetetramine suberate (Bussien Gaillar *et al.*, 1996 ▸) and the co-crystal phenazine–chloroanilic acid (Noohinejad *et al.*, 2015 ▸). In contrast, the hydrogen-bond controlled incommensurately modulated quininium (*R*)-mandelate features no order–disorder transition (Schönleber & Chapuis, 2004 ▸).

**Figure 1:**
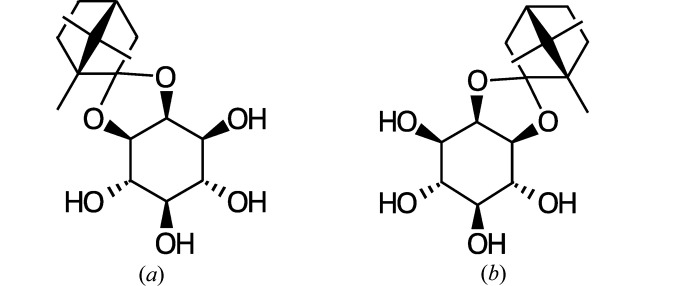
Structural formula of (*a*) **1** and (*b*) its enantiomer described by Gainsford *et al.* (2007) ▸.

In this context, we present the incommensurately modulated structure of the *myo*-inositol-2,3-d-camphor ketal **1** shown in Fig. 1 ▸(*a*). Owing to four hydroxyl groups per molecule, the hydrogen bonding is more complex than in the examples given above. Special attention will be paid to the connectivity of this hydrogen-bonding network in the modulated phase.

**1** is a crucial precursor, since the key challenge in *myo*-inositol chemistry is the selective regio- and stereochemical functionalization of its hydroxyl groups, to mimic decoration patterns of natural inositol derivatives and induce biological activity. Owing to *myo*-inositol being a meso (*i.e* non-chiral) compound, the starting point of synthetic routes towards its derivatives is usually found in some form of desymmetrization. Since its first comprehensive synthesis by Bruzik & Tsai (1992) ▸, **1** has proven itself as a versatile starting point for such endeavors. Combining desymmetrization, protection and the possibility for further selective functionalization, **1** can be synthesized through ketalization of *myo*-inositol with d-camphor dimethylketal and subsequent selective crystallization.

The crystal structure at 170 K of the enantiomer of **1**, *myo*-inositol-1,2-l-camphor ketal [Fig. 1 ▸(*b*)], has previously been described by Gainsford *et al.* (2007) ▸. However, these authors neither report signs of modulation nor a disordered hydrogen-bonding network. They do, however, report a ‘fortuitous’ short contact and the atomic displacement parameters (ADPs) of the hydroxyl O atoms are distinctly enlarged, hinting towards unresolved disorder or overlooked modulation.

## 2. Experimental   

### 2.1. Synthesis and crystal growth   

The synthesis was conducted in accordance with a known literature procedure (Lindberg *et al.*, 2002 ▸). Details of the optimized synthesis and crystal growth procedure as well as hitherto unpublished NMR spectra in CD_3_OD including atom assignment are given in the supporting information (§S1).

### 2.2. Single-crystal X-ray data collection and processing   

The first data collection was performed at 100 K using Mo *K* 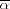 radiation and a CCD detector. Incommensurate satellites were observed, but even with prolonged exposure time reliable intensity data could only be derived for first-order satellites. Therefore, data collection was repeated using Cu *K* 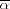 radiation (λ = 1.54184 Å) on a Rigaku Oxford Diffraction XtaLAB Synergy-S diffractometer system equipped with a HyPix-6000HE hybrid photon-counting detector and an Oxford Cryostream 800 plus cooling/heating system. The practically noiseless detector combined with a high-intensity microfocused beam provided the dynamic range (> 10^6^) necessary for the intensity evaluation of higher-order satellites.

A plate-like crystal of **1** was secured to a Hampton Research CryoLoop using a minimum amount of Parabar oil. Diffraction images were acquired with exposure times of 60 s and 110 s at a scan width of 0.5°. The beam divergence was lowered to 3.3 mrad by employing slits, so data could be collected efficiently at 50 mm distance. A data collection strategy to ensure maximum completeness and redundancy was determined using *CrysAlisPro* (Rigaku Oxford Diffraction, 2020*a* ▸). Data processing was performed using *CrysAlisPro* and included a numerical and an empirical absorption correction using the SCALE3 ABSPACK scaling algorithm (Rigaku Oxford Diffraction, 2020*b* ▸).

**Figure 2:**
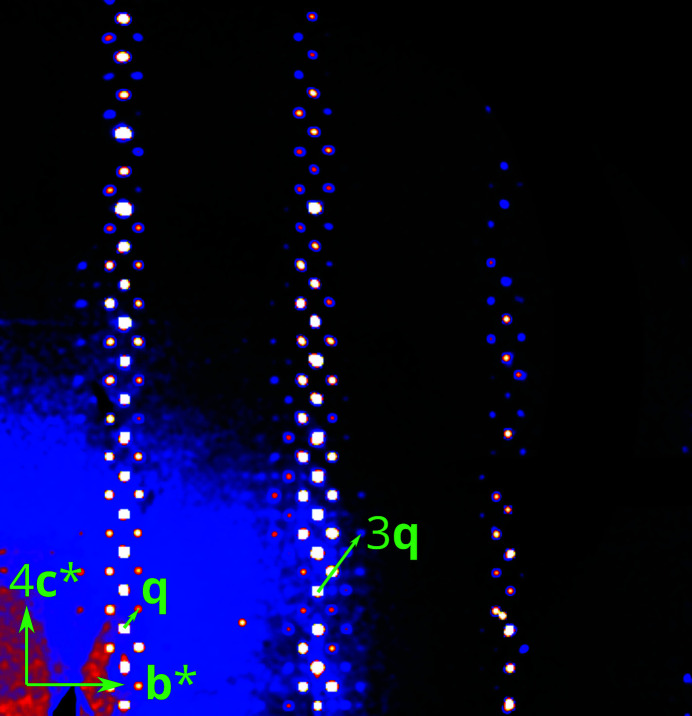
(3*kl*)* plane of reciprocal space of a crystal of **1** reconstructed from 2D detector data. A first- and a third-order satellite are indicated.

Inspection of reciprocal space showed planes of strong reflections corresponding to a monoclinic centered unit cell. With respect to this cell, additional planes of weak reflections normal to b* were observed (Fig. 2 ▸). However, these reflections could not be indexed with a reasonably small super cell as they are satellites of an incommensurately modulated structure. They can be indexed using the **q** = σ_2_ b* + c*/2 modulation wave vector with σ_2_ ≈ 0.15 = 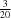. Although very weak, satellites up to the third order were clearly observed and used in subsequent refinements. Moreover, extremely weak reflections were observed at **q**_2_ = σ_2_ b* (note the missing c*/2 term), which indicates either a two-dimensional modulation or the existence of a second domain with a different modulation vector. However, these reflections were too weak for structure refinements and therefore ignored.

More data collection and structure refinement details are compiled in Table 1 ▸. Additional data collections were performed in the 300 K to 400 K range to determine the evolution of the **q** vector.

### 2.3. Structure solution and refinement   

Since **1** was known to be enantiopure by synthesis and the modulation vector is of the form **q** = σ_2_ b* + c*/2, only the *C*2(0σ_2_½) superspace group had to be considered. An initial model was generated using the charge flipping algorithm implemented in *SUPERFLIP* (Palatinus & Chapuis, 2007 ▸). The structure was refined against *F* ^2^ using the *Jana2006* (Petříček *et al.*, 2014 ▸) software.

H atoms connected to C atoms were placed at calculated positions and refined as riding on the parent atoms. Refinements of the hydroxyl H atoms in the incommensurately modulated structure proved to be unstable. Therefore, initial refinements were performed using only main reflections and the space group *C*2 to reliably locate the hydroxyl H atoms. Their positions were refined freely and the isotropic ADPs *U* values were fixed to 1.2× the equivalent isotropic ADPs *U*_eq_ values of the parent O atoms.

The hydroxyl-H atoms will henceforth be designated according to the connected O atoms. For example, the H atom of the O3—H⋯O6 hydrogen bond is named H_3→6_, *etc.* As the CIF format does not allow for special characters, the same H atom is labeled as HO36 in the deposited data. Of the four hydroxyl groups (O3–O6), only the H_6→2_ atom was not disordered. Two H atoms (H_4→4_ and H_5→5_) are disordered about a twofold axis. The other disordered hydrogen bonds are located on the general position (*i.e.* with two distinct H positions per bond). For all disordered H atoms an occupancy of ½ was assumed.

In a next step, satellites up to the third order were included in the refinements. The internal space was well resolved and the 3+1-dimensional *F*_obs_ electron density around the C and O atoms was smooth. Thus, these atoms were refined with harmonic positional modulation functions up to the third order. Attempts using fourth-order modulation functions led to slightly improved residuals on second- and third-order satellites. However, the C—C and C—O distances in these models featured modulations that were deemed physically unrealistic and thus these refinements were dropped. The modulation of the ADPs was modeled with harmonic waves of up to the second order.

**Figure 3:**
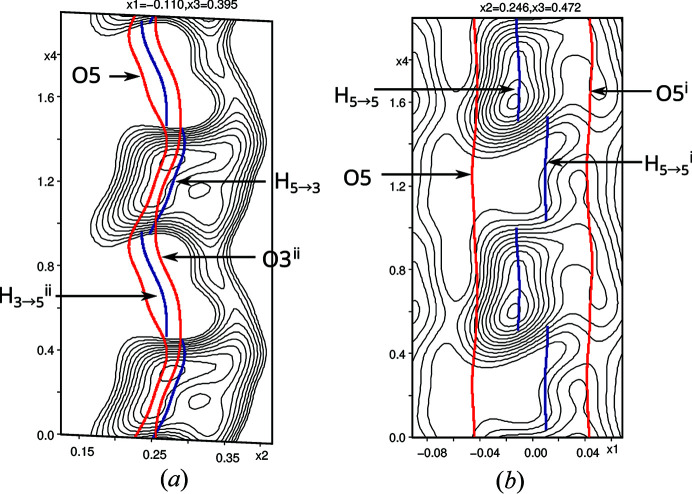
Superspace sections centred around (*a*) H_5→3_ and (*b*) H_5→5_ with 2 Å width. The refined positional displacements are indicated by red (O) and blue (H) lines. Symmetry codes: (i) −*x*_1_, *x*_2_, 1− *x*_3_, *x*_4_ − *x*_3_; (ii) −*x*_1_ − ½, *x*_2_ + ½, −*x*_3_, *x*_4_ − *x*_3_.

The positional modulation of the hydroxyl H atoms was constrained to be identical to those of the parent O atoms and the coordinates in the basic structure were fixed to those derived from the refinement against main reflections, because refinement of the H-atom coordinates generally led to non-convergence, even with distance restraints in place. The occupancy modulation of the hydroxyl H atoms was modeled using crenel functions. The centers of the crenel functions were restrained according to crystal-chemical considerations. Indeed, superspace *F*_obs_ sections centred on the H atoms reflect the choice (Fig. 3 ▸). However, judging the significance of such maps is difficult with X-ray data. It has to be noted that in parts of internal space the hydrogen bonding is ambiguous (equal O—O distances) and possibly disordered. Using X-ray data a reliable determination of the region of disorder is out of the question and therefore usage of discontinuous crenel functions appears justified. The Flack parameter refined to 0.08 (8), thus confirming the expected absolute structure.

## 3. Results and discussion   

### 3.1. Basic structure   

First, a description of the basic structure will be given, which is the hypothetical three-dimensionally periodic structure that is derived from the modulated structure by ignoring the positional, occupational and ADP modulation functions. This differs from the average structure, which is likewise hypothetical and obtained by refining without satellite reflections.

The basic structure has *C*2 symmetry with one crystallographically unique molecule of **1** located in a general position. In principle, it corresponds well to the structure of the enantiomorph of **1** published by Gainsford *et al.* (2007 ▸), though the latter has enlarged ADPs since it corresponds to the average structure and therefore the ADPs reflect the variance of the atomic positions in the modulated structure.

**Figure 4:**
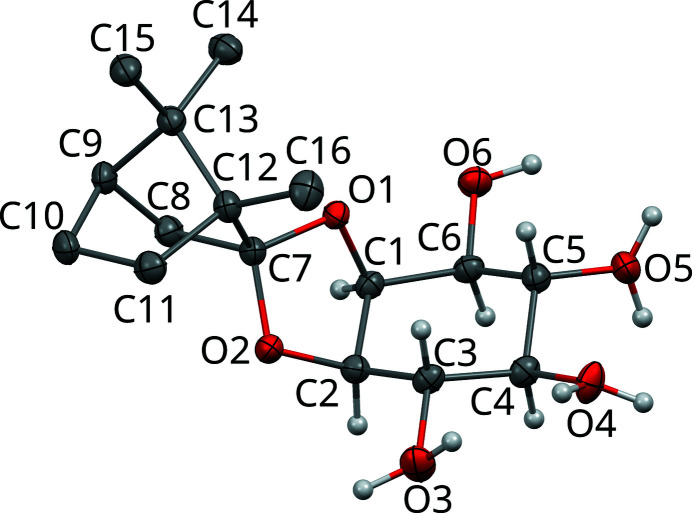
Molecule of **1** in the basic structure with atom-labeling scheme. C (gray) and O (red) atoms are represented by ellipsoids drawn at the 50% probability level, H atoms by spheres of arbitrary radius. H atoms of the bornane moiety are omitted for clarity. For disordered hydroxyl groups (O3, O4, O5), both orientations of the H atoms are shown.

**Figure 5:**
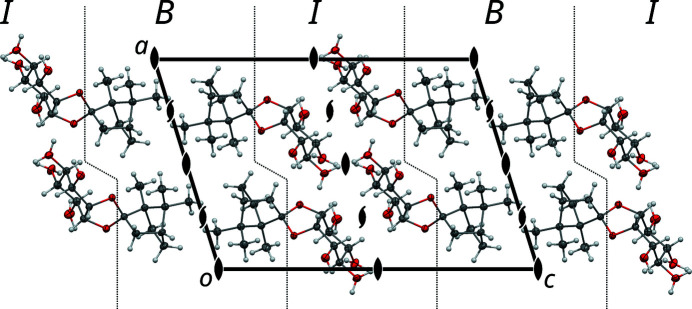
The disordered basic structure of **1** viewed down [010]. Atom colors as in Fig. 4 ▸. Symmetry elements are indicated using the common graphical symbols (Hahn & Aroyo, 2016 ▸). Dotted lines indicate the boundaries between the *B* (bornane) and *I* (inositol) layers. Hydroxyl H atoms are overpopulated owing to disorder.

The molecular structure of **1** and the atom-labeling scheme used herein are depicted in Fig. 4 ▸. The molecules are composed of two distinct parts, on one side a bornane (camphane) spirocycle hydrocarbon (C7–C16) and on the other side a *myo*-inositol sugar-like cyclitol (C1–C6; O1–O6). The *myo* prefix will henceforth be omitted for brevity. As is often observed for structures of organic compounds with distinct hydrophobic (here: bornane) and hydrophilic (here: inositol) parts, these parts congregate into distinct modules. For **1**, they form layers parallel to (001), which will be designated as *B* (for bornane) and *I* (for inositol) as shown in Fig. 5 ▸. Both layers feature *c*121 symmetry (Kopsky & Litvin, 2006 ▸) and can in turn be cut into *half-layers*, which are related by the 2 and 2_1_ operations of the *C*2 space group.

#### 3.1.1. Hydrogen bonding   

All hydrogen bonds in crystals of **1** are inter-molecular. In the basic structure, the hydrogen-bonding network is disordered as summarized in Table 2 ▸. The *D*⋯*A* and H⋯*A* distances fall clearly within the range of *moderate* hydrogen bonds according to the classification of Jeffrey (1997) ▸, which is the range expected for hydrogen bonds in carbohydrates. The longest (and therefore weakest) bond is realized by the non-disordered O6—H⋯O2 hydrogen bond, which is close to the region of *weak* hydrogen bonding.

**Figure 6:**
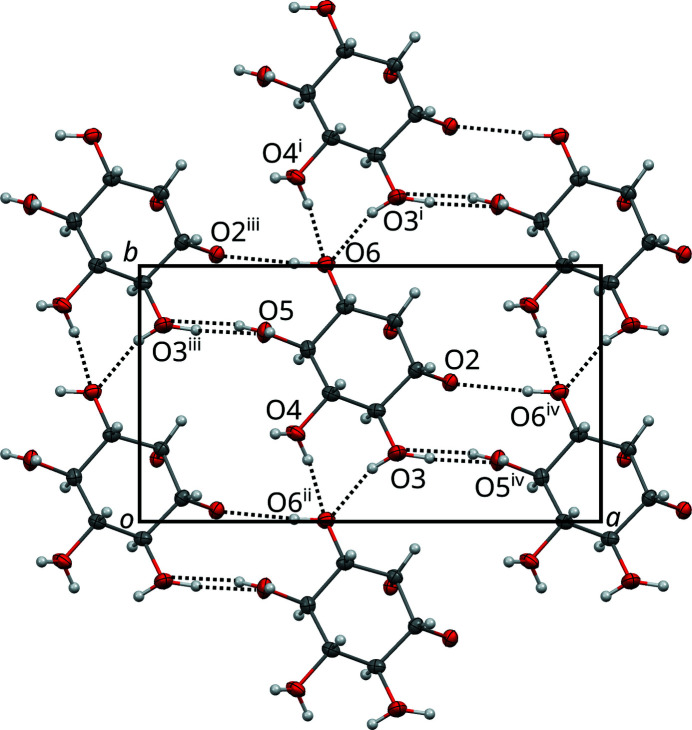
Disordered hydrogen-bonding network in the *I* half-layers of **1** projected on the layer plane (001). Hydrogen bonds are indicated by dotted lines. Hydroxyl groups featuring two H atoms are disordered in a 1:1 manner. Hydrogen atoms without apparent acceptor connect across half-layers. The bornane atoms of the adjacent *B* layers are not shown. Symmetry codes: (i) *t*(0,1,0); (ii) *t*(0,−1,0); (iii) *t*(−½,½,0); (iv) *t*(½,−½,0). Atom colors as in Fig. 4 ▸.

First let us consider only the hydrogen bonding inside the *I* half-layers [Fig. 6 ▸(*a*)]. In such a half-layer, all molecules are related by translations and the hydrogen bonding forms a diperiodic network, whereby the connectivity of the molecules is topologically a square net. This network in turn can be decomposed into two kinds of periodic chains, extending along 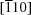 and [010], respectively.

The 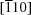 direction features the only non-disordered hydrogen bond, O6—H⋯O2. It is a ‘dead end’ of the network, in the sense that O2 is part of the ketal functionality and therefore does not act as a hydrogen-bond donor. Since it is not disordered in the basic structure, the existence of this bond is not affected by the modulation. Therefore it will not be discussed in the following. Also in the 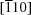 direction, O3 and O5 are connected by a disordered hydrogen bond. In the [010] direction exist disordered O3—H⋯O6 and O4—H⋯O6 bonds.

**Figure 7:**
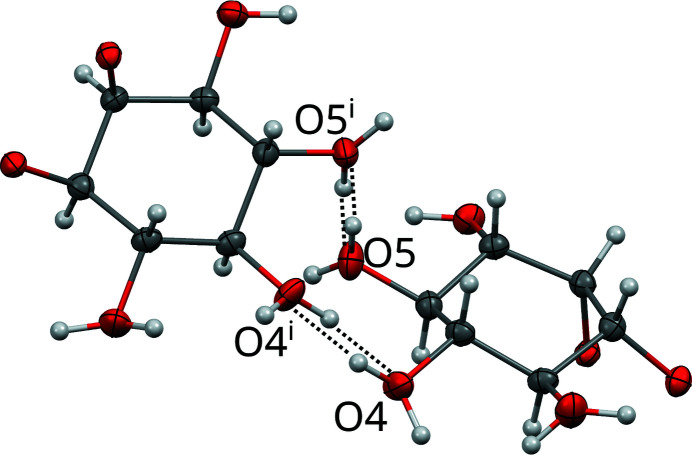
Hydrogen bonds disordered about a 2_[010]_ rotation axis connecting two *I* half-layers of **1** in the basic structure. The viewing direction is distinctly inclined to [010]. Hydroxyl groups featuring two H atoms are disordered in a 1:1 manner. Hydrogen atoms without apparent acceptor connect inside the *I* layers. Symmetry code: (i) 2 0,*y*,½. Atom colors as in Fig. 4 ▸.

Pairs of *I* half-layers are connected by hydrogen bonds to full *I* layers, whereby molecules of **1** are connected via disordered hydrogen bonds between pairs of O4 and O5 atoms. These bonds are disordered about the 2_[010]_ axis in a 1:1 manner (Fig. 7 ▸). Note that since O4 and O5 also feature hydrogen bonding inside the *I* half-layers, the actual occupancies of these disordered bonds might be < ½. However, for the crystal-chemical reasons detailed below, we will assume that the occupancies are at the very least close to ½.

### 3.2. Modulated structure   

#### 3.2.1. Molecular structure   

In general, the C—O and C—C bond lengths vary only slightly with respect to the basic structure [see *t*-plot in the supporting information (§S2)] confirming a reasonable model.

The positional modulation is generally modest, whereby the largest modulation amplitudes are observed for the hydroxyl O atoms, since these atoms are directly involved in the hydrogen bonding. Overall, the **1** molecule behaves as a rigid body, as is observed in many incommensurately modulated molecular structures (Pinheiro & Abakumov, 2014 ▸).

#### 3.2.2. Hydrogen bonding   

In this section chains of hydrogen bonding will be discussed. For brevity, the usual O3—H⋯O5 hydrogen-bond notation will be shortened to O3→O5, which allows for a convenient notation of chains, such as O3→O5→O5. Bonds in the opposite direction will be designated as O3←O5←O5 and the absence of a hydrogen bond between two atoms using a ‘not’ arrow: O3
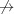
O5. Finally, for disordered bonds or bonds with an undefined direction, a two-sided arrow will be used (*e.g.* O3↔O6).

The domains of definition of the H atoms (*i.e.* the centre of the crenel functions) were derived from O—O distances and crystal-chemical reasoning. In this respect, the O3→O6 and O4→O6 bonds are unique in that they are not fully occupied, yet are directional in the sense that the reverse O3←O6 and O4←O6 bonds do not exist, since O6→O2 is always realized. This is in contrast to the O3↔O5, O4↔O4 and O5↔O5 contacts, which exist in both directions.

Indeed, it appears that the pivotal point of the modulation is the ‘competition’ of the O3 and O4 hydrogen bonds with respect to O6 as acceptor (see Fig. 6 ▸). If both hydrogen bonds were realized simultaneously, a short H⋯H contact of *ca* 1.66 Å as reported by Gainsford *et al.* (2007) ▸ would be realized. Thus, starting from a given **1** molecule, either O3→O6 or O4→O6 will be assumed and the hydrogen-bonding network traced therefrom.

**Figure 8:**
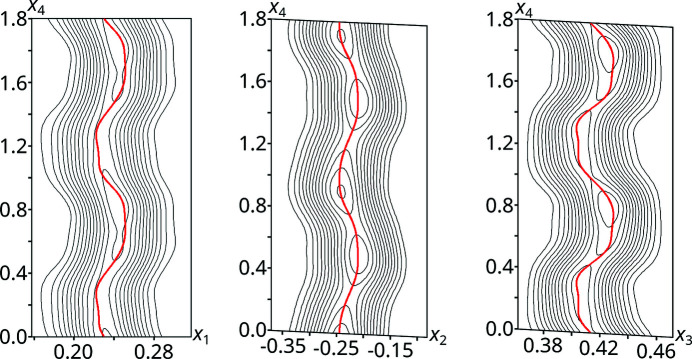
(*x*_1_,*x*_4_) (left), (*x*_2_,*x*_4_) (middle) and (*x*_3_,*x*_4_) (right) superspace sections centered at the position of O3 in the basic structure with 2 Å width. The refined positional displacement is indicated by red lines. Contours are drawn at constant 1 e Å^−3^ levels.

Fig. 8 ▸ shows superspace sections about the O3 atom (O4 features a very similar modulation). For most of the internal space, the *x*_1_ and *x*_3_ coordinates adopt two distinct values, reminding of discontinuous crenel functions. However, the transition between these extremes is smooth and thus every position between these extreme values is adopted at one point.

**Figure 9:**
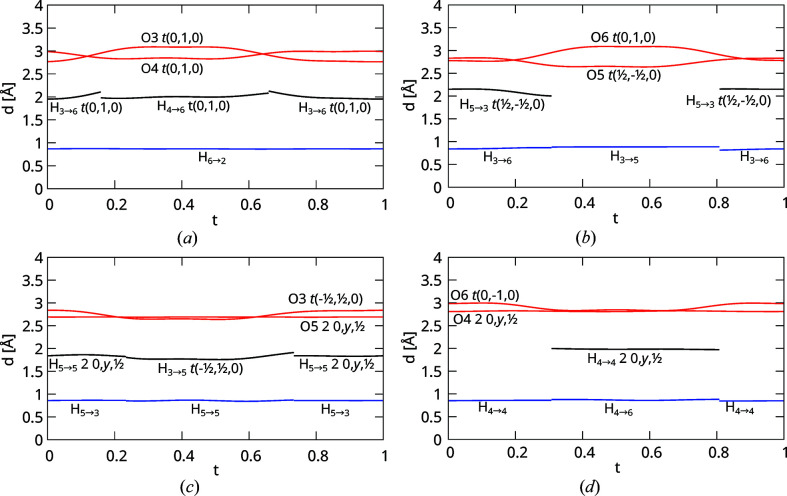
*t*-plot of (red) O—O, (blue) O—H and (black) H⋯O distances of (*a*) O6, (*b*) O3, (*c*) O5 and (*d*) O6.

The resulting O3⋯O6 and O4⋯O6 distances as shown in the *t*-plot in Fig. 9 ▸(*a*). There are two *t*-regions with either O3 or O4 being distinctly closer to O6, respectively. There, the hydrogen bonding can be considered as unambiguous, *i.e.* the hydrogen bonding of the closer atom to O6 is realized. Owing to the continuous modulation functions, there are also two small regions with *d*(O3⋯O6) ≈ *d*(O4⋯O6), where the hydrogen bonding is most likely disordered. However, since the range of disorder cannot be determined on the basis of X-ray data, the domains of definition of both hydrogen bonds were set *approximately* according to the *d*(O3⋯O6) = *d*(O4⋯O6) points. Note that these equidistance points do not delineate regions of precisely one half of the internal space periodicity. However, as will be shown below, owing to the disorder of O4↔O4 and O5↔O5 about a twofold axis in the basic structure, neither O3→O6, nor O4→O6 should be defined for less than half of internal space. Thus, assuming that O3→O6←O4 is never realized simultaneously, the width of the crenel functions must in fact be ½.

Having established the domains of definition of H_3→6_ and H_4→6_, the remainder of the hydrogen-bonding network can be deduced by crystal-chemical reasoning, whereby we will start at the O3 side. Either O3→O6 or O3→O5 is realized. In the former case, O3 acts as acceptor of O5→O3. Thus the domains of definition of H_3→5_ and H_5→3_ can be restrained according to the domain of definition of H_3→6_ as shown in the *t*-plot in Fig. 9 ▸(*b*). There are two distinct regions where either O6←O3←O5 or O6
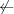
O3→O5 is realized. As previously, both regions are not perfectly realized for half of internal space, which we attribute to disorder in the transition regions.

Continuing tracing the hydrogen bonding, we now arrive at the O5↔O5 bond, which is disordered about a twofold rotation in the basic structure. Here, either an O3→O5→O5 or an O3←O5←O5 fragment is realized and the domain of definition of H_5→5_ can be connected to H_3→5_ and therefore ultimately to H_3→6_. Owing to the *C*2(0σ_2_½) symmetry, by fixing the domain of definition of the H_5→5_ atom to one half of internal space, exactly one of the two possible O5→O5 bonds is realized at every value of *t*. This can be shown by considering the superspace operation of the twofold rotation about 0, *y*, ½, which is 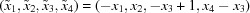. The *t* value of a given atom calculates as 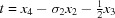. The 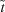 value of the transformed atom then is 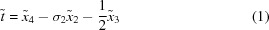


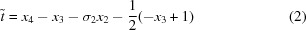


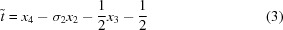


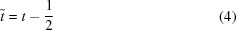
Thus, if an atom is active for a half-period of internal space, its image by the (−*x*_1_, *x*_2_, − *x*_3_ + 1, *x*_4_ − *x*_3_) operation is active for precisely the other half. Note that since O5→O5 and O5←O5 can be active for *at most* half of internal space, O5→O3→O6 has to be active for *at least* half of internal space, as noted above. The resulting distances concerning the O5 atom are shown in Fig. 9 ▸(*c*).

Summarizing, starting with the O6⋯O3 connection, one of two hydrogen bonding chains is realized: 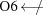
O3→O5→O5→O6→O3 or O6←O3←O5←O5←O3
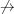
O6.

To the other side of O6, a shorter chain can be traced, since the O4 ↔ O4 bond in the basic structure is disordered about a twofold axis without intermediate atom. Here, the same reasoning as for the O5↔O5 bond applies and therefore either O6
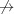
O4→O4→O6 or O6
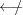
O4←O4←O6 is realized for half of internal space. The corresponding domains of definition and distances are shown in Fig. 9 ▸(*d*).

**Figure 10:**
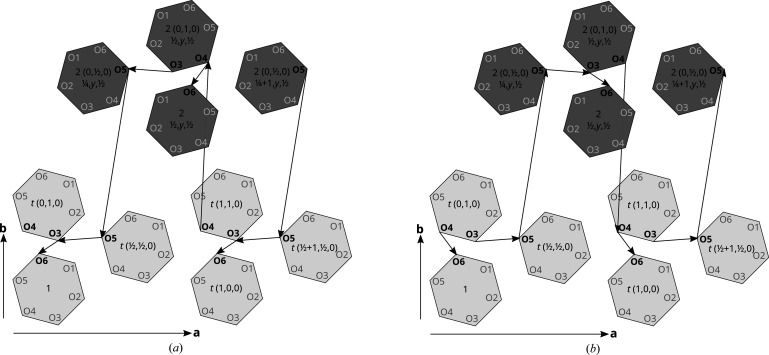
Schemes of infinite chains of **1** molecules connected by hydrogen bonds. Each hexagon represents a molecule. At the center is indicated the operation relating the given molecule to the reference molecule at the bottom left. Light and dark gray hexagons represent molecular orientations related by a 2_[010]_ rotation. Translational symmetry is represented by the position on the graph and is relative to the indicated **a** and **b** basis vectors. Arrows represent hydrogen bonds in direction of the arrow (*i.e.* the acceptor atom is at the end of the arrow).

So far the hydrogen-bonding network was only analyzed locally, leading to two kinds of O6-delimited chain fragments. These two fragments are connected by common **1** molecules, since the final O6 atom connects to either an O3 or an O4 atom of the adjacent molecule. Thus, extended chains are formed, which are schematized in Fig. 10 ▸.

The molecules are marked with their symmetry operation relative to an arbitrary reference molecule (with symmetry 1) in the basic structure. If the O6 atom of that molecule is acceptor of the O3 atom in the adjacent molecule, the chain in Fig. 10 ▸(*a*) is obtained. There are two twofold rotations involved (from O5 to O5 and O4 to O4) leading to a hydrogen-bonding chain with a periodicity of **a** in the basic structure [see molecule marked with *t*(1,0,0)] and containing six **1** molecules up to translation. Since the a* component of the modulation wave vector **q** = σ_2_ b* + c*/2 is zero, the modulated structure is likewise periodic in the [100] direction. In consequence, the hydrogen bonding chains are generally ordered [except in the ambiguous parts of internal space with *d*(O3—O6) ≈ *d*(O4—O6)] and also *periodic*.

If the O6 atom in the reference molecule is connected to O4 instead of O3, the chain schematized in Fig. 10 ▸(*b*) is derived instead. The two chains in Fig. 10 ▸ represent different orientations of the hydrogen bonding network, which can be related by twofold rotation about the 0, *y*, ½ axis.

**Figure 11:**
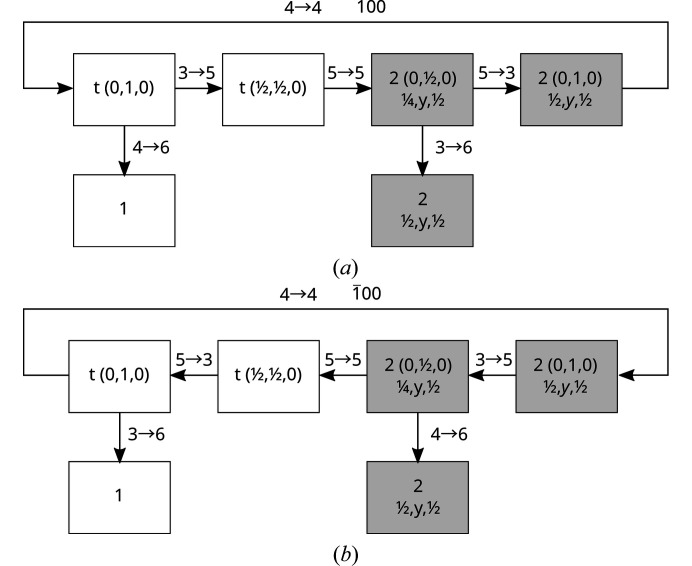
Directed quotient graphs of the hydrogen-bonding chains schematized in Fig. 10 ▸. Each node represents molecules and their equivalents by translation along *n* **a**, 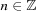. The orientations of molecules represented by white and gray nodes are related by a 2_[010]_ rotation. Arrows represent hydrogen bonds and are labeled with the connected oxygen atoms. A *voltage* of 100 or 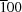 on top of an arrow means that the target node is related to the corresponding node in the 0 < *x* < 1 unit cell by the translation along **a** and −**a**, respectively.

The periodicity in [100] direction allows for a simplification of the extended connectivity networks given in Fig. 10 ▸ by forming their quotient graphs as shown in Fig. 11 ▸. In these graphs each node represents an infinity of molecules that are related by a translation along *n* **a** for all 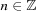. Note that as seen in Fig. 11 ▸, the discussed succession of molecules is, from a topological point of view, not a chain (a linear graph). Rather it represents a linear graph where every second molecule is connected via a O3→O6 or O4→O6 bond to a further molecule. However, these additional molecules must not be ignored since they are the crucial factor determining the orientation of the hydrogen bonding chain.

#### 3.2.3. Modulation of the hydrogen bonding   

Now that the two possible orientations of the hydrogen bonding chains are established, let us focus on the arrangement of these chains in the (001) plane. To complicate matters, each **1** molecule partakes in three such chains, by its O3/O4, O5 and O6 atoms, respectively.

**Figure 12:**
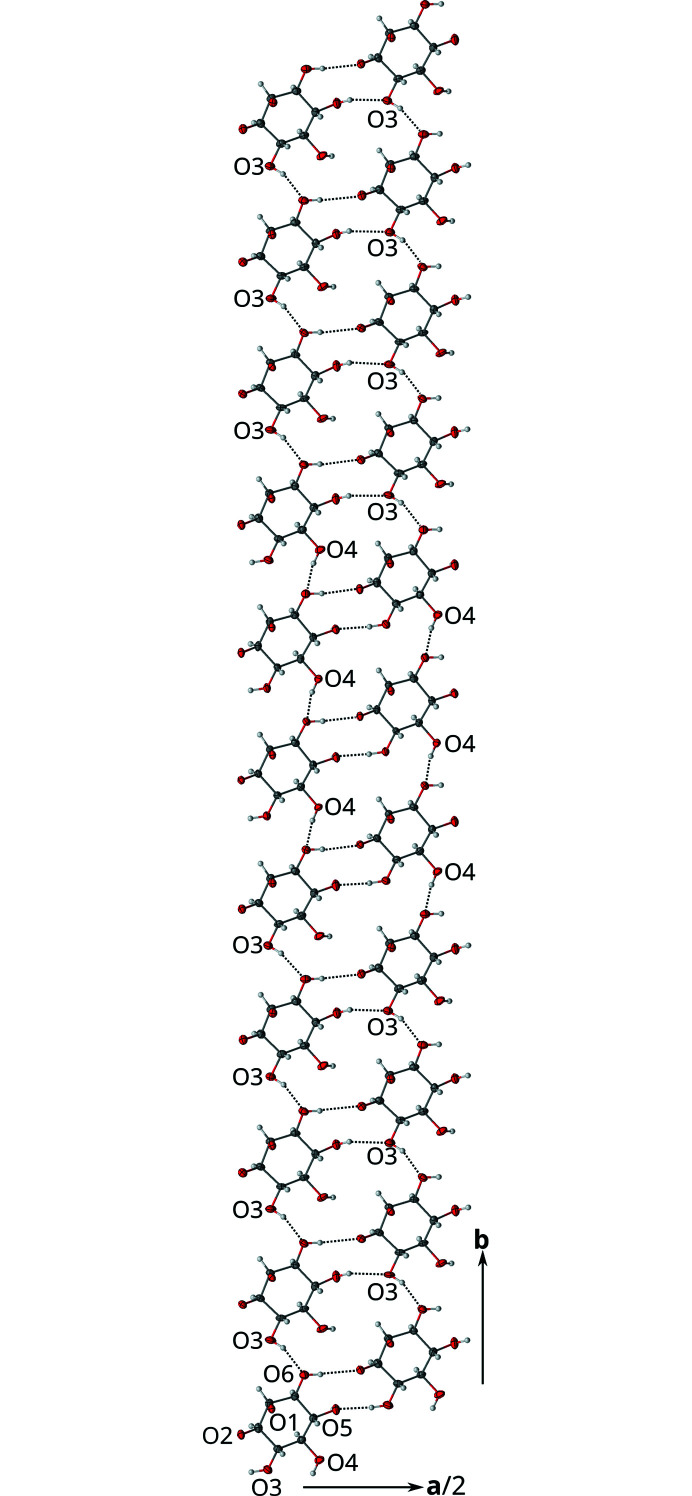
View of an *I* half-layer projected on the layer plane (001) showing the characteristic (left) 3333444333 and (right) 3334443333 succession of chain orientations. Atom color codes as in Fig. 4 ▸.

The chains are stacked in the [010] direction to the hydrogen-bonding network in two ways: On the one hand molecules are related by translation (in the basic structure) along **b**, on the other hand by translation along **a**/2 + **b**/2. Translation along **b** increases the internal coordinate *t* by σ_2_ = 0.1486 (3) ≈ 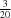. Let us arbitrarily designate a chain with an O3→O6 [Fig. 10 ▸(*a*)] or O4→O6 [Fig. 10 ▸(*b*)] bond in an *I* half-layer by the symbols 3 and 4, respectively. In the other half-layer the other contact is realized. Then, when considering successive **1** molecules in [010] directions, the idealized value σ_2_ = 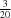 would correspond to an arrangement of chain orientations according to …33334443334444333444… (Fig. 12 ▸). In other words, the chains are arranged in triples and quadruples of the same orientation, whereby after two triples appears a quadruple. Since in the actual structure σ_2_ = 0.1486 (3) < 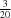 at 100 K, the number of quadruples is slightly higher. Moreover, as has been argued above, at boundaries between three- and four-oriented chains the hydrogen bonding is most likely disordered. The molecules which are translated by the *C*-centering translation **a**/2 + **b**/2 are ‘ahead’ with respect to *t* by σ_2_/2 = 0.0743 (2) ≈ 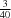. The remarkably ‘slow’ modulation is reflected in first-order satellites which are close to the main reflections.

#### 3.2.4. Bornane layers   

The driving force behind the incommensurate modulation of **1** is certainly the hydrogen bonding. Thus, little is to be said about the crystal chemistry of the intermolecular van der Waals contacts inside the *B* layers, which features only weak modulation amplitudes.

Nevertheless, from a crystallographical point of view, an interesting comment can be made concerning the twofold rotations of the *B* layers when compared to those of the *I* layers. Here, the twofold rotation of the basic structure corresponds to the 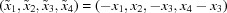 operation. Thus, the *t* value transforms according to [compare with equation (4)]: 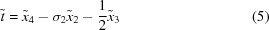


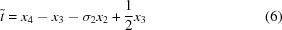


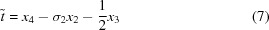


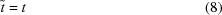
which means that, in contrast to the *I* layers, an atom and its image are active at the same *t* value [see equation (4)]. In other words, these twofold rotations are realized even in the modulated structure, whereas the twofold rotations of the *I* layers are not, which allows for ordered O4→O4 and O5→O5 bonds. This means that whereas in the *C*2 space group of the basic structure the twofold rotations about 0,*y*,0 and 0,*y*,½ are equivalent, they are non-equivalent operations in the *C*2(0σ_2_½) superspace group. Indeed, moving the origin to (0,0,½) leaves the set of operations of the *C*2 group unchanged, but transforms the (−*x*_1_, *x*_2_, −*x*_3_, *x*_4_ − *x*_3_) operation of *C*2(0σ_2_½) into (−*x*_1_, *x*_2_, −*x*_3_, *x*_4_ − *x*_3_ + ½). This alternative setting is designated as *C*2(0σ_2_½)*s* (note the additional *s* symbol). To use the arguably more natural setting *C*2(0σ_2_½), in this work the origin is translated along **c**/2 with respect to the model given by Gainsford *et al.* (2007) ▸.

### 3.3. Thermal properties   

As has been noted in the introduction, incommensurate phases often exist between disordered non-modulated high- and ordered commensurate low-temperature phases (Cummins, 1990 ▸). Yet, even at 430 K, the highest temperature reachable on the employed diffractometer, satellites were still observed.

**Figure 13:**
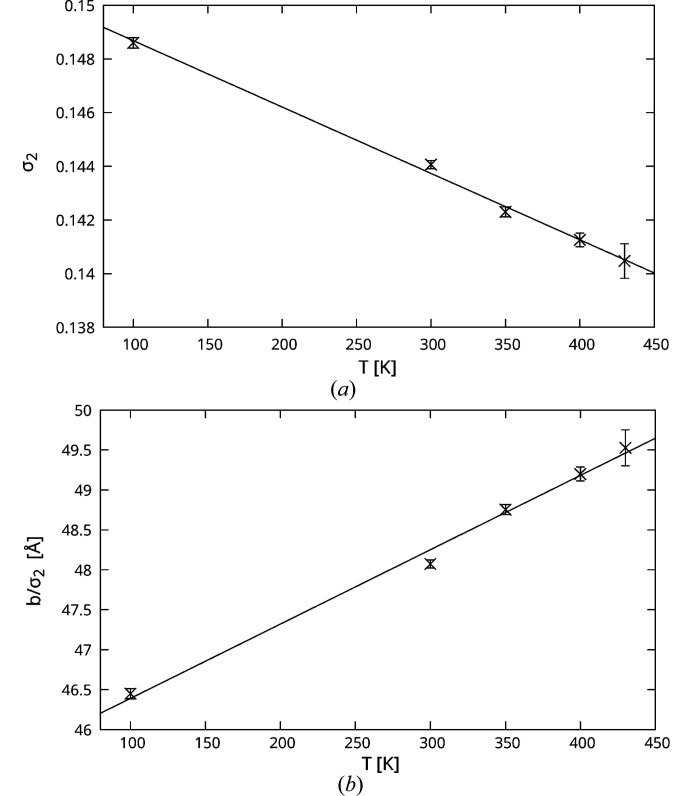
Evolution of (*a*) the σ_2_-component of the modulation wave vector and (*b*) the period *b*/σ_2_ of the modulation wave in [010] direction with temperature. Solid lines are linear least-squares regressions.

The σ_2_-component of the **q** vector decreases with increasing temperature approximately linearly [Fig. 13 ▸(*a*)] from σ_2_ ≈ 0.149 at 100 K to σ_2_ ≈ 0.141 at 430 K (numerical data in the supporting information §S3). This proves that the modulation is dynamic and not commensurate. A decrease of the **q** vector length corresponds to an increase of the period of the modulation wave and thus an increase of the number of quadruples of chain orientations in [010] direction (see §3.2.3). Indeed, for σ_2_ = 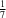 = 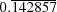, which is realized at ≈ 335 K, the orientation sequence is …3333444…, *i.e.* the number of quadruples reaches the number of triples.

The absolute period of the modulation wave in the [010] direction, which calculates as *b*/σ_2_, increases from ≈ 46.5 Å at 100 K to ≈ 49.5 Å at 430 K, likewise in an approximately linear fashion [Fig. 13 ▸(*b*)] (numerical data in §S3).

Since the modulation is clearly dynamic, *i.e.* the hydrogen bonding continuously reorients in the solid state depending on temperature, one could expect that on heating the structure becomes fully disordered. However, no phase transition could be evidenced using differential scanning calorimetry [supporting information (§S4)].

## 4. Conclusion and outlook   

In this work we presented the incommensurate modulation of a structure with a complex hydrogen-bonding network, which has been missed in a previous structural characterization. Thus, we have shown the importance of revisiting already characterized structures.

Moreover, the example of **1** demonstrates the need for excellent intensity data to unambiguously determine the shape of the modulation functions. Their continuity could only be established by using well-determined weak higher-order satellite intensities.

Nevertheless, owing to the weak contribution of H atoms to X-ray scattering, the structure could not yet be fully characterized with respect to the supposed disordered parts where the chain-orientation flips. Moreover, locating the hydrogen positions indirectly by chemical reasoning is not entirely satisfying. Therefore, neutron diffraction experiments are planned to directly locate these H atoms. Owing to rather small crystals, neutron diffraction data will most likely not provide the resolution (in internal space) that was achieved here with modern X-ray equipment. Thus, only a combination of the two complementary methods will allow unraveling the final secrets of the structure. 

## Supplementary Material

Crystal structure: contains datablock(s) I. DOI: 10.1107/S2052520620015929/dq5049sup1.cif

Structure factors: contains datablock(s) inositol_camphor_ketal. DOI: 10.1107/S2052520620015929/dq5049Isup2.hkl

Synthesis, NMR, thermal properties and molecular conformations. DOI: 10.1107/S2052520620015929/dq5049sup3.pdf

16922EDe7cQ

CCDC reference: 2048290

## Figures and Tables

**Table 1:** Crystal, experimental and refinement data for **1**

	**1**
Crystal data	
Chemical formula	C_16_H_26_O_6_
*M*_r_	314.4
Crystal system, space group	Monoclinic, *C*2(0σ_2_½)
Temperature (K)	100
Modulation wave vector	**q** = 0.1486 (3)b* + c*/2
*a*, *b*, *c* (Å)	12.5887 (7), 6.9000 (2), 18.2523 (7)
β (°)	107.227 (5)
*V* (Å^3^)	1514.31 (12)
*Z*	4
Radiation type	Cu *K* 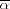
μ (mm^−1^)	0.87
Crystal size (mm)	0.26 × 0.17 × 0.03
	
Data collection	
Diffractometer	XtaLAB Synergy-S, HyPix-6000HE
Absorption correction	Gaussian and empirical
*T*_min_, *T*_max_	0.648, 1
No. of measured, independent and observed [*I* > 3σ(*I*)] reflections	30 326, 17 754, 11 476
*R*_int_	0.022
(sin θ/λ)_max_ (Å^−1^)	0.597
	
Refinement	
*S*	2.09
*R*[*F* ^2^ > 3σ(*F* ^2^)] (%), *wR*(*F* ^2^) (%)	
all reflections	4.44, 14.29
main reflections	3.40, 11.39
first-order satellites	4.91, 13.14
second-order satellite	10.44, 23.64
third-order satellites	15.78, 39.42
No. of reflections [*F* ^2^ > 3σ(*F* ^2^)]	
all reflections	17 754 (11 476)
main reflections	2533 (2529)
first-order satellites	5097 (4985)
second-order satellites	5108 (2703)
third-order satellites	5016 (1259)
No. of parameters	1123
H-atom treatment	H-atom parameters constrained
Δρ_max_, Δρ_min_ (e Å^−3^)	0.28, −0.27
Extinction correction	B-C type 1 Gaussian isotropic (Becker & Coppens, 1974 ▸)
Extinction coefficient	3700 (500)
Flack parameter	0.08 (8)
Absolute structure	8268 of Friedel pairs used in the refinement

**Table 2:** Hydrogen bonds in the basic structure The acceptor molecules are related to the donor molecule by the given symmetry operations. Since the positions of the H atoms were not refined, no esds are given.

*D*—H⋯*A*	Acceptor symmetry	Occ.	*D*⋯H (Å)	H⋯*A* (Å)	*D*⋯*A* (Å)	*D*—H⋯*A* (°)
Inside *I* half-layer						
O3—H⋯O5	*t*(½, −½, 0)	½	0.88	1.86	2.7316 (11)	169
O3—H⋯O6	*t*(0,−1,0)	½	0.84	2.12	2.9274 (9)	163
O4—H⋯O6	*t*(0, −1, 0)	½	0.88	2.04	2.9029 (10)	171
O5—H⋯O3	*t*(−½, ½, 0)	½	0.86	2.08	2.7316 (11)	132
O6—H⋯O2	*t*(−½, ½, 0)	1	0.87	2.20	3.0030 (10)	154
Across *I* half-layers						
O4—H⋯O4	2 0, *y*, ½	½	0.85	1.98	2.8111 (11)	165
O5—H⋯O5	2 0, *y*, ½	½	0.86	1.82	2.6816 (11)	175

## References

[bb30] Becker, P. J. & Coppens, P. (1974). *Acta Cryst.* A**30**, 129–147.

[bb2] Bruzik, K. S. & Tsai, M.-D. (1992). *J. Am. Chem. Soc.* **114**, 6361–6374.

[bb3] Bussien Gaillard, V., Paciorek, W., Schenk, K. & Chapuis, G. (1996). *Acta Cryst.* B**52**, 1036–1047.

[bb4] Canadillas-Delgado, L., Mazzuca, L., Fabelo, O., Rodriguez-Velamazan, J. A. & Rodriguez-Carvajal, J. (2019). *IUCrJ*, **6**, 105–115. 10.1107/S2052252518015026 PMC6327183 30713708

[bb5] Cummins, H. Z. (1990). *Phys. Rep.* **185**, 211–409.

[bb6] Depmeier, W. (1986). *Ferroelectrics*, **66**, 109–123.

[bb7] Gainsford, G. J., Baars, S. M. & Falshaw, A. (2007). *Acta Cryst.* C**63**, o169–o172. 10.1107/S0108270107004416 17339723

[bb8] Gao, Y. & Coppens, P. (1989). *Acta Cryst.* B**45**, 298–303.

[bb9] Hahn, T. & Aroyo, M. I. (2016). *International Tables For Crystallography*, Vol. A, *Space-group symmetry*, 2nd online ed., ch. 2.1.2, pp. 144–148. Chester: International Union of Crystallography.

[bb10] Jeffrey, G. A. (1997). *An Introduction to Hydrogen Bonding* Oxford: Oxford University Press.

[bb11] Kopsky, V. & Litvin, D. B. (2006). Editors. *International Tables For Crystallography*, Vol. E, *Subperiodic groups*, 1st online ed. Chester: International Union of Crystallography.

[bb12] Lindberg, J., Öhberg, L., Garegg, O. J. & Konradsson, P. (2002). *Tetrahedron*, **58**, 1387–1398.

[bb13] Noohinejad, L., Mondal, S., Ali, S. I., Dey, S., van Smaalen, S. & Schönleber, A. (2015). *Acta Cryst.* B**71**, 228–234. 10.1107/S2052520615004084 PMC4383393 25827376

[bb14] Palatinus, L. & Chapuis, G. (2007). *J. Appl. Cryst.* **40**, 786–790.

[bb16] Petříček, V., Dušek, M. & Palatinus, L. (2014). *Z. Kristallogr.* **229**, 345–352.

[bb1] Pinheiro, C. B. & Abakumov, A. M. (2015). *IUCrJ*, **2**, 137–154. 10.1107/S2052252514023550 PMC4285887 25610634

[bb17] Rigaku Oxford Diffraction (2020*a*). *CrysAlisPro*, version 171.41_64.76a. Wrocław, Poland.

[bb18] Rigaku Oxford Diffraction (2020*b*). SCALE3 ABSPACK. Program for absorption correction. Wrocław, Poland

[bb19] Schmidt, V. H. (1987). *Ferroelectrics*, **72**, 157–173.

[bb20] Schönleber, A. & Chapuis, G. (2004). *Acta Cryst.* B**60**, 108–120. 10.1107/S0108768103027605 14734850

[bb21] Tabatabaee, M., Poupon, M., Eigner, V. P., Vaněk & Dušek, M. (2018). *Z. Kristallogr.* **233**, 17–25.

