# Supplementary material for: The role of hydrogen bonding in the incommensurate modulation of myo-inositol camphor ketal
Source: Acta Crystallogr B Struct Sci Cryst Eng Mater. 2021 Jan 22;77(Pt 1):83–92. doi: 10.1107/S2052520620015929 (PMC7941279; doi:10.1107/S2052520620015929)
Supplement: Supplementary file 3 [file b-77-00083-sup3.pdf]

STRUCTURAL SCIENCE
CRYSTAL ENGINEERING
MATERIALS

Volume 77 (2021)

Supporting information for article:

The role of hydrogen bonding in the incommensurate modulation of *myo*-inositol camphor ketal

Viktor Savic, Felix Eder, Christian Göb, Marko D. Mihovilovic, Christian Stanetty and Berthold Stöger

The role of hydrogen bonding in the incommensurate modulation of *myo*-inositol camphor ketal

Viktor Savic,[a] Felix Eder,[b] Christian Göb,[c] Marko D. Mihovilovic,[a] Christian Stanetty[a] and Berthold Stöger [d]*

[a]*Institute of Applied Synthetic Chemistry, TU Wien, Getreidemarkt 9/163, A-1060 Vienna Austria,* [b]*Institute of Chemical Technologies and Analytics, TU Wien, Getreidemarkt 9/164, A-1060 Vienna Austria,* [c]*Rigaku Europe SE, Hugenottenallee 167, 63263 Neu-Isenburg Germany, and* [d]*X-Ray Center, TU Wien, Getreidemarkt 9/164, A-1060 Vienna Austria. E-mail: bstoeger@mail.tuwien.ac.at*

incommensurate modulation; hydrogen bonding; X-ray diffraction; sugar chemistry

S1. Synthesis

D-*camphor dimethyl ketal:*

In a 250 mL round bottom flask *D*-camphor (15.5 g, 101 mmol, 1.0 equiv.) was dissolved in trimethyl orthoformate (77.5 mL, 712 mmol, 7.0 equiv.) and methanol (15.5 mL) was added to the solution. Under moderate stirring, sulfuric acid (0.15 mL, 3 mmol, 0.03 equiv.) was slowly added, resulting in a cloudy solution, which cleared up after a few minutes. After 36 h 8% starting material was still present according to NMR-analysis upon micro-work up. Nevertheless, the reaction mixture was neutralized through addition of sodium methoxide (325 mg, 6.0 mmol, 0.06 equiv.) and concentrated in *vacuo*, resulting in a suspension to which toluene (75 mL) was added.

Upon filtration and concentration under reduced pressure, a pale yellow liquid was obtained (19.4 g, 85$\%_{wt}$ purity), containing the product, toluene (10 mol%) and starting material (10 mol%) and used as-is.

Ketalization:

Conc. sulfuric acid (252 μL, 4.7 mmol, 0.08 equiv.) was added to a solution of *myo*-inositol (4.48 g, 8.88 mmol, 1.0 equiv.) and camphor dimethylketal (14 g of crude mixture corresponding to 60 mmol of pure ketal, 6.7 equiv.) in dry DMSO (49 mL). The mixture was stirred for 3 h at 75 °C, after which HPTLC indicated full conversion of the starting material (chloroform:methanol = 4 : 1). Upon neutralization with triethylamine (1.68 mL, 12 mmol, 0.2 equiv.), dimethyl sulfoxide was removed in *vacuo* (2.0 mbar, 75 °C) resulting in a total weight of 22 g.

Reactive crystallization and isolation:

Chloroform (75.5 mL), methanol (4.8 mL), water (1.51 mL) and *p*-toluene sulfonic acid (16.8 mg) was added and the mixture was stirred for 17 h during which time a colorless solid precipitated. The solid was collected by filtration and washed with chloroform (2 $\times$ 60 mL) and dried in *vacuo* to yield the crude product as a colorless solid (6.31 g). ^{13}C-NMR showed the desired diastereomer to be the major species out of three detected compounds.

Purification by trituration:

The crude material was triturated in hot methanol (130 mL) containing triethylamine (1%) for 1 h at reflux temperature. The solid was collected by filtration, washed with chloroform (2 $\times$ 60 mL) and dried in *vacuo* to yield **1** in good (diastereomeric) purity according to ^{1}H and ^{13}C-NMR (see below).

Yield 2.7 g (37% based on *myo*-inositol). Platy crystals were grown by slow evaporation of a methanol solution at room temperature.

NMR data:

^{1}H-NMR (600 MHz, CD_3OD) δ (ppm) = 0.86 (s, 3H), 0.89 (s, 3H), 1.04 (s, 3H), 1.18–1.25 (m, 1H), 1.40 (td, J = 12.2, 4.7 Hz, 1H), 1.50 (d, J = 13.0 Hz, 1H), 1.70–1.79 (m, 1H), 2.01–2.05 (m, 1H), 2.05–2.11 (m, 1H), 3.13 (dd, J = 10.1, 9.2 Hz, 1H), 3.44 (dd, J = 10.1, 7.2 Hz, 1H), 3.52 (t, J = 9.5 Hz, 1H), 3.69 (dd, J = 9.7, 4.3 Hz, 1H), 3.78 (dd, J = 7.2, 5.5 Hz, 1H), 4.27 (dd, J = 5.5, 4.2 Hz, 1H). ^{13}C-NMR (151 MHz, CD_3OD) δ (ppm) = 10.4, 20.9, 21.2, 28.0, 30.6, 46.5, 46.6, 52.6, 71.8, 73.8, 75.2, 77.4, 77.7, 77.9, 118.9. ^{13}C-spectra in pyridine-$d6$ were identical with the literature data (Bruzik & Tsai, 1992). Original NMR data including atom assignments are given in Figs. S1, S2.

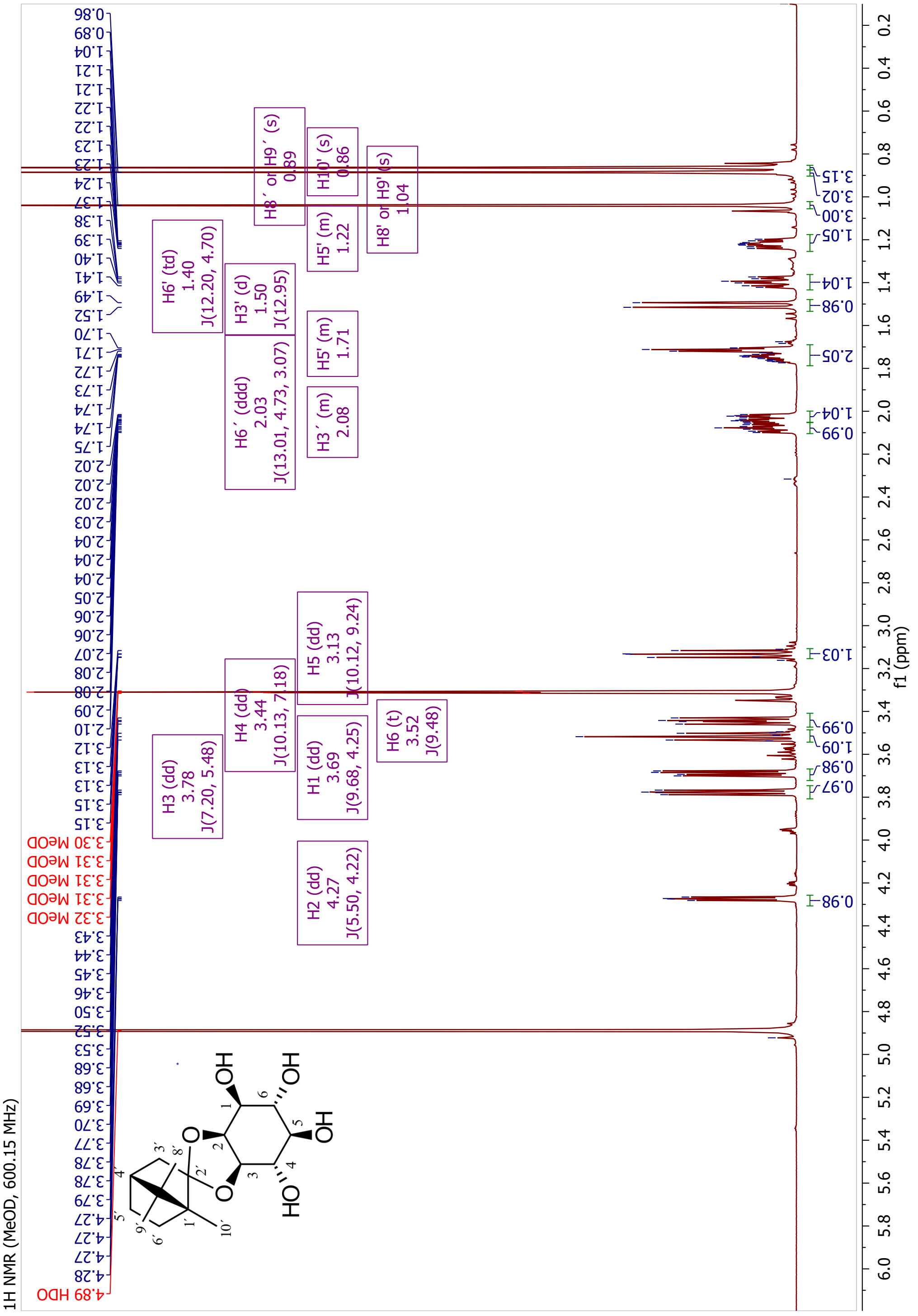

Fig. S1. ^{13}H-NMR of **1** with atom assignments.

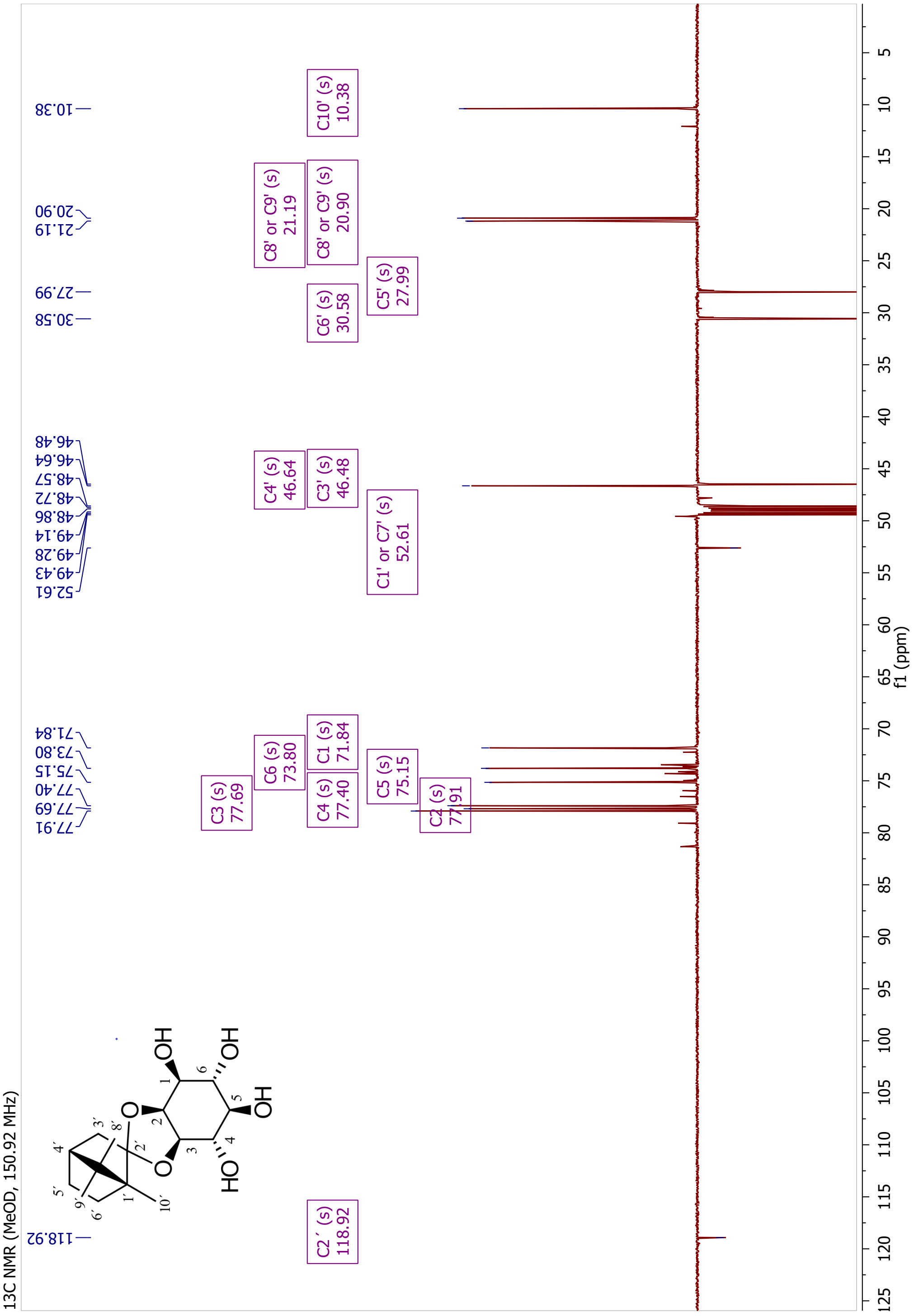

Fig. S2. ^{13}C-NMR of **1** with atom assignments.

S2. Modulation of the molecule

The C—C and C—O distances are given as t-plots in Fig. S3. As expected, the C—C distances in the strained bornane moiety feature a greater spread with the shortest and longest bond both involving the quaternary C12 atom.

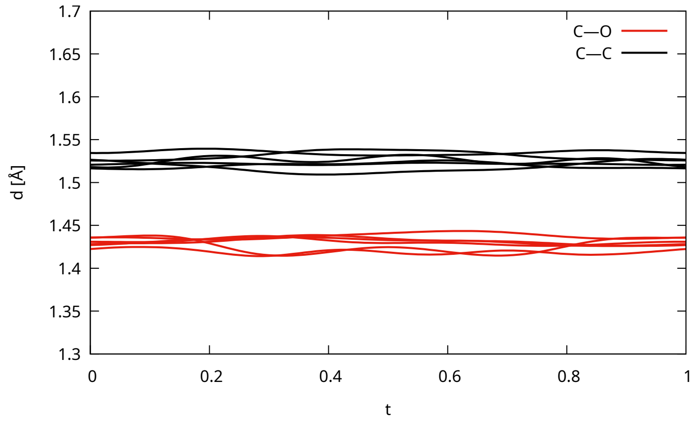

(a) inositol

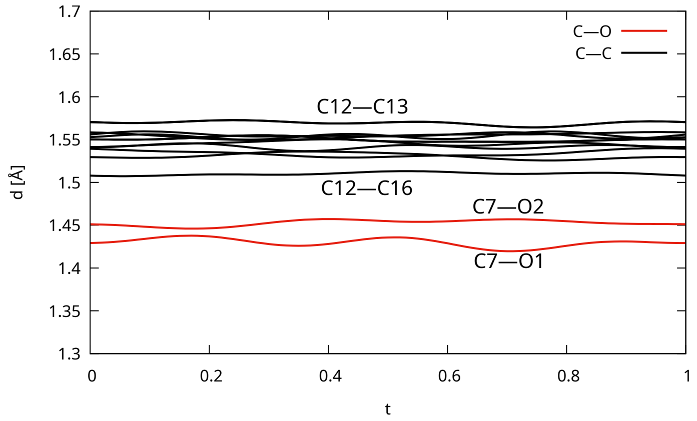

(b) bornane

Fig. S3. t-plots of the (red) C—O and (black) C—C bond lengths of (a) the inositol and (b) the bornane moiety in **1**.

S3. Evolution of the q-vector with temperature

Table S1 gives the temperature dependence of the σ_2-component of the **q**-vector as well as the absolute period of the modulation weave in [010] direction in numerical form.

Table S1. *Evolution of the σ_2-component of the modulation wave vector and the period b/σ_2 of the modulation wave in* [010] *direction with temperature.*

T (K)	σ_2	b/σ_2 (Å)
100	0.1486 (2)	46.45 (6)
300	0.1441 (2)	48.07 (5)
350	0.1423 (2)	48.75 (6)
400	0.1416 (3)	49.20 (9)
430	0.1405 (6)	49.5 (2)

S4. Thermal analysis

In search of an order/disorder phase transition to a disordered non-modulated phase, a combined DSC/TGA analysis of microcrystalline **1** powder was performed using a NETZSCH STA 449 F1 Jupiter simultaneous thermal analyzer in the 300–525 K temperature range under Ar atmosphere in an Al crucible with pierced lid.

A finely powdered sample of **1**, which was single-phase according to X-ray powder diffraction, was subjected to DSC measurements in the 300 to 525 K temperature range (Fig. S4). The endothermic melting effect has an onset of 505 K, which corresponds precisely to the melting point determined using an OptiMelt melting point apparatus. It is overlain by a broad endothermic effect starting at ca. 480 K, which we attribute to decomposition, since it is accompanied by a mass loss of ca. 1.7 %. There are no signs of other effects of either the first or the second order and thus no clear evidence of a disordering of the structure up to its melting point (with decomposition).

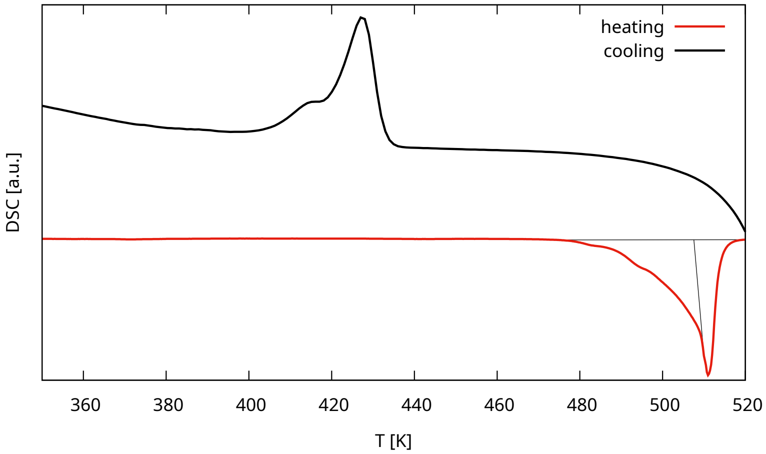

Fig. S4. DSC trace of microcrystalline **1** powder on (red) heating and (black) cooling. The onset of the melting effect is indicated. Exothermic up.

On cooling, recrystallization occurs at a strongly depressed temperature of ca. 435 K, which is probably due to partial decomposition and a general tendency of sugars and sugar-like polyols to form supercooled liquids.

References

Bruzik, K. S. & Tsai, M.-D. (1992). *J. Am. Chem. Soc.* **114**, 6361–6374.
